# Disparities in access and association between access to critical facilities during day-to-day and disrupted access as a result of storm extreme weather events

**DOI:** 10.1016/j.heliyon.2023.e18841

**Published:** 2023-08-01

**Authors:** Flavia Ioana Patrascu, Ali Mostafavi, Arnold Vedlitz

**Affiliations:** a Urban Resilience.AI Lab, Zachry Department of Civil and Environmental Engineering, Texas A&M University, College Station, TX, 77843, USA; b Public Policy, Bush School of Government and Public Service, A&M University, College Station, TX, 77843, USA

**Keywords:** Equitable access, Critical facilities, Resilience, Disasters

## Abstract

This study examines the relationship between households' access to critical facilities day-to-day and during weather-related extreme events. Despite a robust understanding of both day-to-day access and access during disasters, the interplay between the two remains unclear. To bridge this knowledge gap, we propose a novel empirical approach, using a Texas statewide household survey (N = 810). The survey evaluates day-to-day and past events access, exploring the experiences of respondents during multiple recent disasters, rather than focusing on a specific hazard. Using correlation analysis, we examined various access-related factors such as day-to-day trip duration, alternative trip duration, and loss of access during past events. Additionally, we evaluated the association between access-related factors and sociodemographic characteristics such as income, ethnicity, and urban status. The results indicate: (1) daily trip duration to critical facilities is associated with disrupted access during storm events, and (2) disparities persist during both day-to-day times and during extreme events. These results bring new insights to the existing body of knowledge on day-to-day access and access during disasters. The findings provide scientifically grounded evidence to city managers and planners, emphasizing the need for equitable distribution of facilities to enhance access to essential facilities both in daily life and during extreme weather-related events.

## 1 Introduction

Critical facilities are at risk of disruption by natural hazards [[1], [2], [3]]. Maintaining access to these facilities is crucial for preserving daily life [4]. However, the effects of disasters vary among diverse subpopulations. Urban areas experience segregation in terms of racial groups and socioeconomic status, and even more so when disaster impacts are considered [5]. Furthermore, research indicates that mitigation efforts to reduce the impacts of hazards, such as climate adaptation, are creating increasingly significant disparities among urban counties in the US [6]. Therefore, it is crucial to acknowledge disparities in examining the relationship between day-to-day access and disrupted access during extreme weather events caused by storms.

Access and accessibility have received growing attention in city planning in the last decades [7]. The measurement of access has been examined from the perspectives of urban planning, facility location optimization, and public health [[8], [9], [10]]. However, it is important to distinguish between the two terms. Access refers to the possibility of reaching a place, or services [11]. While, accessibility measures the easiness with which a place can be reached via certain means of transportation [12]. Furthermore, Kelobonye, Zhou [13] find accessibility to indicate the build's environment efficiency and spatial equity. Most studies examine access from the characteristic of proximity by using spatial distance [8,14]. Limitations of this approach come from how distance is defined. Often distances are based upon length measurements of large areal units, which accounts for Euclidian distance rather than transportation network distance [8]. For example, the same Euclidian distance in an urban setting could represent a significantly longer travel time to reach a facility than in a rural one. Other approaches have examined travel time as a complementary measure to distance [14,15]. Using travel time as a measure of access captures differences such as: (1) means of transport: walking, public transport, or personal vehicle; or (2) a combination of the means of transport including the transit time; or (3) accounting for personal choice of path.

Innovative data-driven research proposes detecting access to critical facilities through either intelligence location data [16,17] or infrastructure-based sensors monitoring road traffic [18]. While these methods have benefits in terms of the easiness and rapidness of data collection, it also presents several limitations and biases. To address these challenges, recent research studies point to the benefits of using survey empirical driven methods for understanding both access [19] and disparities [20,21].

Access to critical facilities during normal conditions and during storm-related events has been thoroughly investigated. Nonetheless, there is a scarcity of information regarding the association between the two. This study aims to bridge this gap by presenting a theoretical framework exploring the relationship between day-to-day (steady-state) access to critical facilities and disrupted access during storms. The framework will be validated through a statewide survey conducted in Texas. To address these gaps, this study aims to: (1) examine the association between a community's day-to-day access to critical facilities and access during extreme storm-related events; and (2) investigate the relationship between access and the population's sociodemographic characteristics.

Our study aims to fill these gaps by answering the research questions related to the extent of the association between day-to-day access to critical facilities and disrupted access during storm events and the degree of disparities in access among different subpopulations during both normal conditions and storm-related events. We will approach these questions by developing a theoretical framework and 5 specific research questions to examine (see Table 2) the access to critical facilities and disparities. Further we will test our assumptions through a household survey in Texas, using correlation analysis for validation. The study will be comprised of the following sections: (1) Literature Review (Section 2), (2) Methods (Section 3), (3) Case Study (Section 4), (4) Findings (Section 5), and (5) Concluding Remarks (Section 6).

**Table 1:** Key concepts used throughout the paper.

Concept	Description
Access	By access, we refer to the ability to reach certain critical facilities [11].
Day-to-day access	By day-to-day access, we mean the “normal” access, mainly the steady-state type of access [22,23], without the stress of an extreme weather-related event. This type of access refers to the time duration needed to reach a certain critical facility. This type of access assumes the use of personal means of transportation and the preferred route available.
Disruption in access	By disruption in access, we refer to a difficulty or constraint to access certain services [22]. A disruption can either be an increased travel time or the total loss of access as a result of a storm-related event.
Increased travel time	We define the increased travel time as the extra time needed for an individual to reach a critical facility during a storm event, as suggested by Neutens, Delafontaine [22].
Loss of access	We consider the loss of access to be an incapacitation to reach [24] a certain critical facility as a result of a storm-related event. The loss of access could be a result of secondary causes such as road inundations, high winds or power outages.
Resilience	In this study, resilience refers to the ability of systems (ranging from infrastructure to community systems) to resist and bounce back from the shock of a hazard [25].
Access to critical facility indicators	Access is often measured through proximity and can be quantified through time duration and distance [22]. Another important aspect of access is the ability to reach a place (accessible/not accessible).
Trip time duration	Trip time duration represents the time required for an individual to reach a destination by using the fastest available route and personal transportation, as defined by Neutens, Delafontaine [22].
Accessibility	Accessibility is a measure of how easily a location can be reached using a specific mode of transportation [12].
Disparity	Disparities are great differences in the way one particular or certain subpopulations access certain critical facility [26]. The disparities can occur before, after and/or during certain storm-related extreme events [24].

**Table 2:** Study's specific research questions.

No.	Specific research questions and approach
**RQ1**	**To what extent is day-to-day access to critical facilities associated with an increased travel time due to a storm-related event?** Testing the relationship between the normal trip duration to reach a critical facility and the time it took to reach the same type of facility during a previous storm event.
**RQ2**	**To what extent would day-to-day access to critical facilities exacerbate the loss of access due to a storm-related event?** Testing the relationship between the normal trip duration to a critical facility and the loss of access to the same type of critical facility during a previous storm event.
**RQ3**	**To what extent would vulnerable subpopulations need more time to reach critical facilities during day-to-day?** Testing the relationship between the normal trip duration to a critical facility and people's sociodemographic characteristics.
**RQ4**	**To what extent would populations in rural areas need more time to reach alternative critical facilities due to a storm-related event?** Testing the relationship between the increased travel time to a critical facility as a result of a storm-related past event and the sociodemographic characteristics of people.
**RQ5**	**To what extent are vulnerable subpopulations more prone to losing access to critical facilities due to a storm-related event?** Testing the relationship between the loss of access to critical facilities as a result of a storm-related past event and the sociodemographic characteristics of people.

## 2 Literature review

Access to critical facilities is essential for maintaining daily life activities during normal conditions and disasters. Such facilities, including grocery stores, pharmacies, gas stations, healthcare facilities, social services, workplaces, and schools, play a crucial role in fulfilling human rights, such as the Right to Adequate Living Standard outlined in the United Nations Article 25 [27]. Additionally, access to critical facilities is a key component of the United Nations Sustainable Development Goals (SDGs) [28], including food, water, healthcare, and education. Despite this, many communities still lack access to essential services like grocery stores, schools, and medical care, even in the absence of disasters [9]. Logan and Guikema [9] emphasize the importance of access to these facilities when assessing community resilience and call for ensuring equitable access as a key factor in overall resilience.

The existing literature lacks a human-centered approach to addressing societal risks [29], which translates into the absence of empirical evidence of a household's susceptibility to access infrastructure disruptions. To address this gap and integrate societal impacts into resilience, it's crucial to first understand household vulnerability to service losses [30,31]. Access to critical infrastructure has been identified as a valid proxy for household vulnerability to service losses. Studies have explored access to critical facilities and infrastructure through the lens of individual facilities such as transportation [14,32], healthcare [[33], [34], [35]], and grocery stores [24]; and by examining multiple critical facilities together [34,[36], [37], [38]].

### 2.1 -to-day access to critical facilities

Day-to-day access to critical facilities varies based on various factors such as the day of the week, time of the day, personal habits (proximity versus choice) and proximity to the critical facilities [22]. Recent studies emphasize the importance of considering both distance and time in assessing access to these types of facilities [9,22]. Banke-Thomas, Wong [39] compared the emergency services travel time data with the cost friction surface approach using Open-Source Routing Machine and Google Maps and found that these methods underestimated the precision of the travel time. Therefore, measuring people's travel duration to critical facilities could provide a more accurate understanding of their access.

People across the world face disruptions in access to vital facilities such as healthcare, grocery stores, schools, and others, even without the presence of hazards [9,40,41]. This is often due to the unequal distribution of facilities between central urban and marginal areas. To address this issue, urban planning methods such as polycentric planning have been proposed as a solution [42]. Ensuring this type of planning will mitigate the existence of “deserts” in accessing critical facilities and provide overall better access. The term ‘desert’ was adopted from the concept of ‘urban food deserts' [43]. Based on this context, an area is considered as a ‘food desert’ if over two-thirds of its population resides more than a mile away from affordable, nutritious food [44,45]. However, the difficulties in access to critical facilities are not uniform among all subpopulations, with vulnerable groups often experiencing the greatest challenges. For example, patterns in income [5,36] and race or ethnicity [46] have been identified as factors contributing to disparities in access to critical facilities such as healthcare. Unfortunately, even in the absence of disasters, many individuals from these vulnerable subpopulations reside in areas characterized as food deserts or healthcare deserts, or lack access to other critical facilities [9]. Our research aims at gathering empirical evidence on the relationship between day-to-day access and disrupted access and the disparities in access among vulnerable subpopulations. Integrating the association of access and disparities will provide a holistic approach to assessing access deserts and could support planners in informed decision-making for resource allocation and new infrastructure prioritization.

### 2.2 Disruptions in access as a result of a storm related event

Previous studies report disruptions in access to critical services as a result of storm related events as varying among different subpopulations [30,31,37]. For instance, low-income and ethnic minorities experience greater losses in access due to transit disruptions [30,47]. Hence, it is essential to understand potential equity issues in access to critical facilities [47] in order to address them both day-to-day and during a disaster context [2,10,14,15]. Recent literature has emphasized the importance of equitable access by addressing access disparities, vulnerability, and capacity of various subpopulations [30,34,24,37,38,48] during storm related disasters while focusing on different aspects related to access.

Our empirical study advances the existing knowledge through empirical evidence regarding the relationship between day-to-day access and disrupted access to critical facilities during storm events. Access to critical facilities during disasters have been associated with people using the nearest option to reach critical facilities [49] and therefore we assume when people answer the questions related to the time required to reach critical facilities in a storm-related past event, they refer to the fastest and shortest route by using a private means of transport.

## 3 Methods

**Fig. 1:**
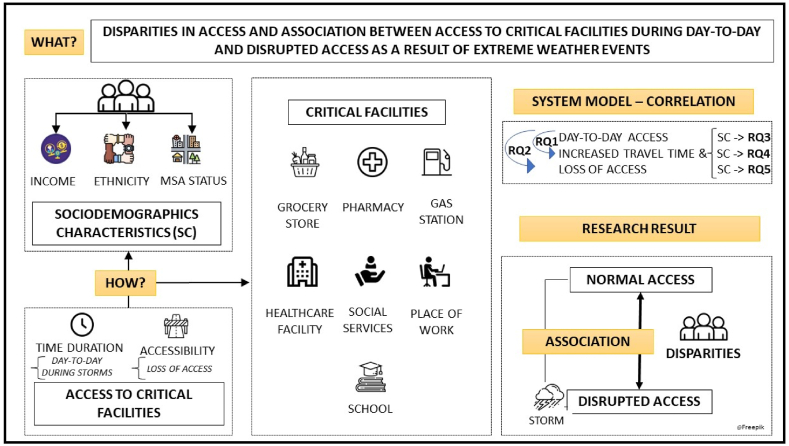
Conceptual framework.

The research framework is outlined in Fig. 1. We conduct an empirical study by analyzing the following access indicators: (1) time duration to access critical facilities day-to-day and in storm-related extreme weather; and (2) accessibility to critical facilities. An in-depth description of these indicators and all concepts addressed throughout this study are described in Table 1. The proposed study focuses on how various subpopulations recover from multi-hazards. In this context, the hazards refer to extreme weather events associated with storms. These storm-related extreme weather events impact through their compound effects population's access, attributable to the simultaneous presence of strong winds and heavy rainfalls, under conditions of either extreme heat or cold. Such conditions often lead to significant disruptions. These disruptions in access can manifest directly, due to flooding, high winds, or fallen trees; or indirectly, as a result of power outages or lack of alternative routes. The objective of our study is to monitor how the day-to-day access of the population to various critical facilities is affected by these disruptions and to understand the unique recovery processes undertaken by different subpopulations faced with these challenges. We suggest that access to various critical facilities, as illustrated in Fig. 1, is of utmost importance. These facilities are grocery stores, pharmacies, gas stations, healthcare facilities, social services, places of work, and schools. Access to these facilities is deemed important as households with better access to such facilities can achieve a higher level of resilience and therefore can better withstand disaster impacts [24]. We use correlation analysis, as shown in Fig. 1 - System Model and Table 2, to address the relationship between day-to-day access to critical facilities and disrupted access due to storms, and the underlying disparities. To assess disparities in access to critical facilities, we associate access with sociodemographic characteristics. For determining vulnerable subpopulations, we considered the following sociodemographic information as relevant: income level, ethnicity, and metropolitan statistical area (MSA) status.

Based on the concepts outlined in Table 1, our analysis evaluates people's accessibility to critical facilities by examining the time required to reach them day-to-day and during a past storm event and the related disparities in access by understanding people's sociodemographic information. As the goal of the study is to evaluate the association between access to critical facilities during day-to-day times and a disaster, we implemented the survey during the post-event recovery phase of the Disaster Risk Management Cycle.

Further, as outlined in Table 2, the research questions are described. Research question RQ1 investigates the relationship between daily access to critical facilities and increased travel time during a storm. RQ2 enquires the loss of access to critical facilities during a past storm event. Research questions RQ3, RQ4, and RQ5 examine disparities in access to critical facilities among vulnerable populations, such as low-income and minority households.

## 4 Case study

### 4.1 Study context

Recent years have seen Texas endure several extreme weather events, including Hurricane Harvey in 2017 and Winter Storm Uri in 2021 causing substantial economic damages and disruptions in access to critical facilities. For example, according to Lindner and Fitzgerald [50], Hurricane Harvey alone caused disruptions and damages both as a result of direct flooding or indirect consequences. Overall, over 4.7 million people were impacted, 36 lost their lives, 60,049 people were rescued through government rescue actions and tens of thousands more by local civilians. On top of the disruptions to human life, the extreme weather event resulted in inaccessibility of roads, power outages and over 300,000 cars flooded across Harris County. However, this survey is a result of the high exposure to weather related extreme events, both winter storms and hurricanes. In light of various events that have previously caused hardship, this study does not examine a specific hazard event, but rather examines a geographic area (State of Texas) and the experience of respondents in multiple recent storm-related hazard events. With Texas facing frequently extreme weather events, this region presents as an ideal location for examining our research questions.

### 4.2 Survey design

**Fig. 2:**
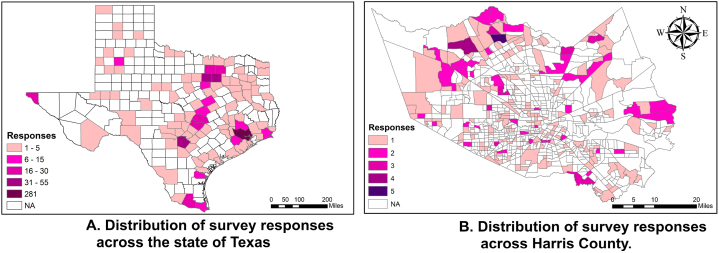
Survey responses distribution. 2 A (left panel): Distribution of survey responses across the state of Texas. 2 B (right panel): Distribution of survey responses across Harris County.

The survey was deployed from April 26, 2021, through May 6, 2021 b y Ipsos Public Affairs (Ipsos) on KnowledgePanel®, a probability-based web panel designed to be representative of the United States population. The target population consisted of adults aged 18 and older residing in the state of Texas as well as an additional oversample of adults aged 18 and older in Harris County, Texas, county in which Houston is located. Panel members chosen for the study were sent an email invitation requesting them to fill out the survey at their earliest possible convenience. The survey consisted of 147 question-items. A total of 1585 surveys were fielded. The completion rate of 51% yielded 810 completed surveys. The median survey completion time was 15.65 min. The survey was conducted in English. In addition to the survey variables from the main interview, Ipsos’ standard demographic profile variables and a series of data processing variables created by Ipsos were provided. The samples collected consist of 810 responses (N = 810) distributed across the state of Texas, with a high density of 281 responses from Harris County, as shown in Fig. 2.

The household survey examined access to critical facilities: average day-to-day trip duration, the additional trip duration due to a storm-related past event, loss of access due to a storm-related past event, the respondents' sociodemographic information and location. Although the survey did not collect information on the transportation modes used by the respondents, it is important to note that personal vehicle ownership rates in the surveyed census tracts are generally high. According to the American Community Survey, less than 6% (5.2%) of households in these tracts do not have a personal vehicle [51]. Therefore, it is safe to assume the respondents used personal means of transportation. However, this limitation may affect the generalizability of the findings to households without access to personal vehicles.

### 4.3 Data and measurements

The data collected during the survey has two dimensions: access dimension and sociodemographic dimension.

Table 3 summarizes the survey questions related to the time needed to reach critical facilities during day-to-day and during a storm related extreme event which occurred in the last 5 years.

**Table 3:** Survey questions measuring household access (N = 810).

Access measurement	Survey Question	Data type	Mean	Standard deviation
Day-to-day access	About how many minutes does it typically take to travel one way between your home and the closest:	Quantitative: Minutes		
	Grocery store		8.45	6.92
	Pharmacy		8.21	7.60
	Gas station		5.39	4.53
	Healthcare facility		13.57	10.93
	Social services		6.45	13.11
	Place of work		11.50	16.52
	School		3.22	6.99
Increased travel time	During a past storm-related event in the last 5 years, if available, how many minutes did you need to travel one way to get to an alternative:	Quantitative: Minutes		
	Grocery store		8.87	12.43
	Pharmacy		8.29	11.69
	Gas station		6.66	10.04
	Healthcare facility		14.21	21.46

Table 4 presents the results of the question on households' loss of access to a critical facility as a result of a storm-related event in the last 5 years. The response format is binary, with options of either “yes” or “no.” The frequency and percentage of households that reported loss of access are shown in the table.

**Table 4:** Survey questions measuring household loss of access (N = 810).

Access measurement	Survey Question	Data type	Frequency (Yes)	Percentage (%) (Yes)
Loss of access	During any past floods in the last 5 years, did you lose access to any of the following facilities or services that you used before the flood?	Binary: Yes (=1) No (=0)		
	Grocery store		187	0.230
	Pharmacy		166	0.204
	Gas station		165	0.203
	Healthcare facility		170	0.209
	Social services		71	0.087
	Place of work		99	0.122
	School		84	0.103

Table 5 summarizes the sociodemographic characteristics of the survey participants, including their income, ethnicity, and metropolitan statistical area (MSA) status. The income level was recorded on an ordinal scale and recoded into a 7-point scale, with 1 representing the lowest income and 7 representing the highest. The survey data reveal a good representation of income diversity, as shown in Table 5. Participants were classified into two ethnic groups: White (= 1) and Non-White (= 2). Of the respondents, 57.2% identified as White, demonstrating a well-distributed diversity of ethnic groups. The MSA status indicates whether the respondents live in a metropolitan statistical area (MSA) as defined by the US Office of Management and Budget Core-Based Statistical Area [52], with 1 representing metro areas with populations ≥50,000 and 2 representing non-metro (suburban or rural) areas with populations <50,000. While the fact that the ratio of 93.7%–6.3% in favor of metro areas could be considered a limitation, it is worth noting that a greater percentage of the population in Texas lives in metro areas, and therefore, the percentages accurately reflect the population distribution.

**Table 5:** Sociodemographic characteristics of household survey (N = 810).

Household subdomain group	Survey response and encoding	Frequency	Percentage (%)
Income	Less than $10,000 (= 1)	31	0.038
	$10,000 to $24,999 (= 2)	78	0.096
	$25,000 to $49,999 (= 3)	147	0.181
	$50,000 to $74,999 (= 4)	151	0.186
	$75,000 to $99,999 (= 5)	130	0.16
	$100,000 to $149,999 (= 6)	141	0.174
	$150,000 or more (= 7)	132	0.163
Ethnicity	White, non-Hispanic (= 1)	463	0.572
	Black, non-Hispanic (= 2)	88	0.109
	Other, non-Hispanic (= 2)	30	0.037
	Hispanic (= 2)	211	0.26
	2+ races, non-Hispanic (= 2)	18	0.022
MSA Status	Metro (as defined by U.S. OMB Core-Based Statistical Area) (= 1)	759	0.937
	Non-Metro (= 2)	51	0.063

### 4.4 Statistical analysis

A correlation analysis was performed using the R studio software. The significant level for the results was set at p < 0.01, p < 0.05, and p < 0.1, according to current statistical practices. It should be noted that the idea of establishing a fixed threshold at p < 0.05 for the level of significance has been rejected by statisticians, who consider other characteristics, such as the purpose of the analysis and data sample size [53,54]. Given the specific purpose and sample size of the current analysis, a threshold of p < 0.01, p < 0.05, and p < 0.1 was deemed appropriate.

To examine the relationship between day-to-day access to critical facilities and disruptions caused by storm events, we analyzed its correlation to day-to-day access (RQ1) and to disruptions in access (RQ2) by using the Pearson correlation test [55]. This test was selected because the type of data (continuous variables) used for RQ1 and RQ2. Additionally, we investigated the correlations between access-related indicators and sociodemographic characteristics (RQ3, RQ4, and RQ5) to examine disparities in access. The Spearman's correlation test was used for addressing RQ3 and RQ5, as the data are on an ordinal scale with larger dimensions [55]. The smaller sample size for research question RQ4 necessitated the use of the Kendall's correlation test (Field, 2009).

## 5 Findings

The correlation coefficients for the access indicators (Table 6) range from 0.41 to 0.84, while the coefficients for the correlation between access indicators and the sociodemographic characteristics (Table 7) range from 0.08 to 0.21. While these correlation coefficients may be considered weak in some fields [56,57], it is important to note that different cutoff points are commonly used in the literature [56] when interpreting correlations. Given that our data consists of subjective opinions, we use a classification system that considers a correlation coefficient less than 0.1 as weak [56], between 0.1 and 0.3 as moderate, between 0.3 and 0.6 as strong, and greater than 0.6 as very strong.

**Table 6:** Association between access to critical facilities during normal times and disrupted access due to a storm-related event.

RQ	Correlation analysis	Variable name	Texas state sample		
			p-value	Coefficient	Number of non-zero samples
RQ1	Day-to-day access correlated with increased travel time. (Pearson's test)	Grocery store	0.0001^a^^,^^b^^,^^c^	0.61^d^	112/810
		Pharmacy	0.0001^a^^,^^b^^,^^c^	0.41^d^	94/810
		Gas station	0.0001^a^^,^^b^^,^^c^	0.55^d^	93/810
		Healthcare facility	0.0001^a^^,^^b^^,^^c^	0.47^d^	97/810
RQ2	Day-to-day access correlated with loss of access. (Pearson's test)	Grocery store	0.2072	0.07	392/810
		Pharmacy	0.0760	0.09	390/810
		Gas station	0.2449	0.06	390/810
		Healthcare facility	0.1812	0.07	388/810
		Social services	0.0001^a^^,^^b^^,^^c^	0.84^d^	167/810
		Place of work	0.0001^a^^,^^b^^,^^c^	0.76^d^	223/810
		School	0.0001^a^^,^^b^^,^^c^	0.78^d^	186/810

a – significant p-value <0.1.

b – significant p-value <0.05.

c – significant p-value <0.01.

d – estimate >0.1.

**Table 7:** Disparities in access to critical facilities.

RQ	Correlation analysis	Variable name	Texas state sample						
			Income		Ethnicity		MSA status (Urban/rural)		Number of non-zero samples
			p-value	Coefficient	p-value	Coefficient	p-value	Coefficient	
RQ3	Day-to-day access correlated with sociodemographic characteristics (Spearman's test)	Grocery store	0.0002^a^^,^^b^^,^^c^	−0.14^d^	0.1464	0.06	0.2924	0.04	795/810
		Pharmacy	0.0008^a^^,^^b^^,^^c^	−0.12^d^	0.2199	0.05	0.0709^a^	0.07	787/810
		Gas station	0.2026	−0.05	0.8734	−0.01	0.6237	0.02	791/810
		Healthcare facility	0.0001^a^^,^^b^^,^^c^	−0.18^d^	0.0821^a^	0.07	0.4466	0.03	788/810
		Social services	0.5477	−0.04	0.4433	−0.05	0.3076	−0.06	318/810
		Place of work	0.0118^a^^,^^b^^,^^c^	0.13^d^	0.6465	−0.03	0.9603	−0.01	416/810
		School	0.3146	−0.06	0.0382^a^^,^^b^^,^^c^	0.11^d^	0.3638	0.05	356/810
RQ4	Increased travel time correlated with sociodemographic characteristics (Kendall's test)	Grocery store	0.9883	−0.01	0.6793	0.04	0.0817^a^^,^^b^^,^^c^	0.15^d^	113/810
		Pharmacy	0.5603	−0.06	0.4215	−0.08	0.1677	−0.13	96/810
		Gas station	0.9785	0.01	0.5015	−0.07	0.2437	0.11	96/810
		Healthcare facility	0.5599	0.06	0.1522	−0.13	0.0975^a^^,^^b^^,^^c^	0.15^d^	98/810
RQ5	Loss of access correlated with sociodemographic characteristics (Spearman's test)	Grocery store	0.0044^a^^,^^b^^,^^c^	−0.15^d^	0.5609	0.03	0.0001^a^^,^^b^^,^^c^	−0.21^d^	397/810
		Pharmacy	0.1798	−0.07	0.5287	−0.04	0.0009^a^^,^^b^^,^^c^	−0.17^d^	398/810
		Gas station	0.2897	−0.06	0.9816	0.01	0.0011^a^^,^^b^^,^^c^	−0.17^d^	399/810
		Healthcare facility	0.2074	−0.07	0.5311	0.04	0.0002^a^^,^^b^^,^^c^	−0.2^d^	397/810
		Social services	0.0433^a^^,^^b^^,^^c^	−0.16^d^	0.0394^a^^,^^b^^,^^c^	0.16^d^	0.0114^a^^,^^b^^,^^c^	−0.2^d^	174/810
		Place of work	0.6537	0.03	0.5539	0.04	0.0101^a^^,^^b^^,^^c^	−0.17^d^	232/810
		School	0.9558	0.01	0.0668^a^^,^^b^^,^^c^	0.14^d^	0.004^a^^,^^b^^,^^c^	−0.21^d^	192/810

a – significant p-value <0.1.

b – significant p-value <0.05.

c – significant p-value <0.01.

d – estimate >0.1.

### 5.1 Research question 1 (RQ1): day-to-day access to critical facilities is associated with an increased travel time due to a storm-related event

The results of the Pearson correlation test (Table 6) indicate moderate to strong correlations between day-to-day access to critical facilities and the alternate duration in case of a storm-related event for grocery stores, pharmacies, gas stations, and healthcare facilities. On average, the increase in time to reach these facilities was 77.63% for grocery stores, 77.71% for pharmacies, 119.67% for gas stations, and 81.67% for healthcare facilities. These facilities are particularly important for vulnerable populations and the results emphasize the significance of considering day-to-day access as an indicator of community resilience during disasters [4]. These findings have important implications for city planners, decision-makers, public officials, and emergency managers. They should consider examining objective access to critical facilities and adopt strategies, such as equitable facility distribution, to improve day-to-day access. The results also suggest the importance of identifying areas with poor access, which can result in “access deserts”. To anticipate increased difficulties in accessing critical facilities during extreme weather events, decision-makers and emergency managers should integrate day-to-day access into the preparedness phase. This will allow them to identify areas that are most prone to experiencing disruptions based on day-to-day access to critical facilities. Improving day-to-day access not only enhances the quality of life for residents but also reduces disrupted access during extreme weather events. By reducing disrupted access to facilities, we can support protective actions, such as preparedness (proposed by Li and Mostafavi [58]) as well as recovery [48]. These findings highlight the need for objective examination of day-to-day access to critical facilities and the adoption of strategies to improve access for all populations.

### 5.2 Research question 2 (RQ2): day-to-day access to critical facilities can exacerbate the loss of access due to a storm-related event

The Pearson correlation test results presented in Table 6 reveal a significant correlation between day-to-day access to critical facilities and the disruption of this access during a past storm event. Our analysis found a significant correlation between the daily accessibility to critical facilities and the loss of access due to a storm (as indicated by increased travel time to alternative facilities). The loss of access to critical facilities during a storm can have a significant impact on populations, as they are unable to reach these facilities for a period of time. Moreover, our findings suggest a very strong correlation between the day-to-day duration of accessing social services, places of work, and schools and the loss of access to these facilities during a storm event. This correlation indicates that individuals who have longer commutes and those who live farther away from their places of work and schools are more vulnerable to losing access to these facilities during a storm. These results emphasize the importance of identifying areas that are prone to experiencing disrupted access to critical facilities during storm events. By identifying and addressing areas with poor day-to-day access, decision-makers and emergency managers can reduce the vulnerability of populations during storm events. Additionally, improving daily access to critical facilities not only enhances the quality of life for residents but also facilitates protective actions and facilitates recovery during and after a storm event.

### 5.3 Research question 3 (RQ3): vulnerable subpopulations need more time to reach critical facilities day-to-day

The results of the Spearman's correlation test, as presented in Table 7, indicated a correlation between the daily access to critical facilities and the sociodemographic characteristics of the population. The findings suggest that vulnerable subpopulations, such as those with lower income and the minority status, need to spend more time on average to reach critical facilities like grocery stores, pharmacies, healthcare facilities, and their place of work. This correlation is supported by prior research, such as the studies conducted by Fan, Jiang [4], Coleman, Esmalian [30], Esmalian, Coleman [24], which have reported similar disparities in access. By adding this result to the one from RQ1, we can understand how day-to-day disparities in access contribute to disparities impacts of disasters among low-income groups. During times of crisis, these populations face additional difficulties accessing critical facilities due to longer travel times, exacerbating their already disrupted access. The results also revealed that higher-income populations tend to spend more time reaching their place of work. This could be explained by a variety of factors, including a preference for a good school district, a desire to live in a better neighborhood, or a personal choice to live in suburbs, resulting in a longer commute. This result, when considered alongside those from RQ2, highlights the increased vulnerability of higher-income populations to work-related disruptions due to extended commutes during normal times.

### 5.4 Research question 4 (RQ4): populations in rural areas need more time to reach alternative critical facilities due to a storm-related event

The results of a Kendall's correlation test (Table 7) indicated a relationship between the duration of time required to reach alternative grocery stores and healthcare facilities and the metropolitan statistical area (MSA) status (urban, suburban, or rural). The findings revealed that rural households require a longer time to access alternative facilities in the event of a storm. As access to alternative facilities within a reasonable travel duration is an indicator of redundancy in the availability of critical facilities [24] the results indicate that populations in rural areas are less resilient in the face of disrupted access. This could result in greater hardship and slower recovery following extreme weather events. Therefore, this finding highlights the significance of examining the access to critical facilities in rural areas where there is limited redundancy. To enhance the resilience of rural populations in the face of future extreme weather events, efforts should be made to retrofit existing facilities and roads. This can improve access to critical facilities and accelerate recovery following extreme weather events.

### 5.5 Research question 5 (RQ5): vulnerable subpopulations are more prone to losing access to critical facilities due to a storm-related event

The results of Spearman's correlation test (Table 7) revealed significant association between the loss of access to critical facilities due to storm-related events and various sociodemographic characteristics of population. Specifically, a significant correlation was identified between access loss to grocery stores and social services and household income. The findings indicate that lower-income households are more likely to experience loss of access to these critical facilities. Additionally, we found a significant association between the loss of access to social services and schools and the minority status. This suggests minority subpopulations may be more vulnerable to losing access to these critical facilities in the event of a hazard. Another significant finding highlights the association between loss of access to various critical facilities, including grocery stores, pharmacies, gas stations, healthcare facilities, social services, places of work, and schools, and the urban or rural status of households. Unlike the results reported in RQ4, the study found that households in urban areas are more susceptible to losing access to these critical facilities during extreme weather events. These findings emphasize the importance of considering both urban and rural areas in regional hazard mitigation and disaster risk reduction plans. It is essential to proactively identify and reduce the physical vulnerability of infrastructure and facility distribution that could result in loss of access to critical facilities during future extreme weather events.

## 6 Concluding remarks

Our study's findings concerning the associations between day-to-day and disrupted access and disparities contribute new insights to the field. The effects of infrastructure service disruptions vary among different vulnerable sub-populations as they are not uniform across all groups as indicated by Dong, Esmalian [34] and others [37,59,60]. However, our results are consistent with some literature findings, which suggest that vulnerable subpopulations often struggle with access, even without the occurrence of a hazard (as highlighted by Logan, Williams [8]). We've observed expected recovery inequities for low-income subpopulations following a disaster, a point also noted by Peacock, Van Zandt [61]. Additionally, Coleman, Esmalian [30] also reported disruption inequalities in transportation for both minorities and low-income subpopulations. However, our study sets itself apart from existing research in several ways. Firstly, our focus is on a broad state-wide survey during the post-recovery phase, without linking to a specific event. Instead, we've considered a five-year period during which several storm-related extreme events occurred. Secondly, our methodology and survey tool evaluate population access to critical facilities, differing from studies that focus on household service disruptions [30] or a blend of household services and healthcare access, like medication and healthcare facilities [62]. Lastly, our study extends current understanding of equitable access to critical facilities. We do this by exploring the relationship between everyday access and the disruptions during extreme weather-related events. Additionally, we scrutinize disparities at the household level. To our knowledge, no prior studies have empirically investigated this relationship. Therefore, we believe these unique aspects of our research significantly enrich the field's collective understanding. The research investigated the association between day-to-day access to crucial facilities and disruption caused by storm-related events. Significant empirical evidence suggests poor access to critical facilities during normal times could be a bellwether to disruptions during a weather-related event. The analysis revealed: (1) day-to-day access to essential facilities, such as grocery stores, pharmacies, gas stations, and healthcare facilities, influences the availability of alternative facilities during a storm event; and (2) extended travel time to these facilities in normal times exacerbates the disruption of access during a storm event for social services, workplaces, and schools. These findings highlight the need for acknowledging the areas with limited access during mitigation and emergency response planning. The findings have imperative implications for designing and planning effective strategies to improve access to critical facilities during both day-to-day and disasters. To anticipate difficulties in access or loss of access to critical facilities, decision-makers and emergency managers should take into account day-to-day access when planning, and identify areas most vulnerable to loss of access based on their daily level of access to critical facilities.

However, the present study has some limitations. For example, there is an imbalance in the sample size between metro and non-metro areas, with a higher representation of metro subpopulation. This might be explained by the higher response rate of metro respondents or by the fact that there is a higher number of people residing in metro areas in Texas. This study and its findings open new venues for future research. For example, future studies can further investigate the associations between day-to-day and disrupted access to critical facilities and evaluate the role of infrastructure inequality and facility distribution patterns. To address potential disparities, it would be intriguing to compare our proposed correlation approach with an ordinary linear regression and examine whether these models can include control variables to eliminate the impact of confounding factors on the outcomes. In addition, future studies can examine the relationship between facility access and population preparedness and recovery in disasters to answer the following research questions: to what extent day-to-day access levels affect the ability of populations to prepare for an impending hazard event? Or to what extent disrupted access during disasters could delay population recovery?

The findings provide empirical evidence of how disruptions in access to critical facilities occur disproportionately among subpopulations during extreme weather-related events. For example, low-income subpopulations experienced higher difficulties in access to the grocery store during normal time and loss of access as a result of a weather-related event. Also, the findings showed the disproportionate loss of access among populations of rural areas compared with those living in urban settings. This result highlights the importance in examining access to critical facilities in rural areas where there is little redundancy. The existing facilities and roads that provide access to those facilities should be retrofitted to improve the resilience of rural areas to future extreme weather events.

## Ethic statement

The research study conducted in this present work followed the established ethical guidelines of the Institutional Review Board Amendment ID: IRB2021-0117 M with investigator Arnold Vedlitz. The survey received approval from the Human Research Protection Program (HRPP) committee.

## Author contribution statement

Flavia Patrascu: Analyzed and interpreted the data; Contributed reagents, materials, analysis tools or data; Wrote the paper.

Ali Mostafavi: Conceived and designed the experiments; Performed the experiments; Analyzed and interpreted the data; Contributed reagents, materials, analysis tools or data; Wrote the paper.

Arnold Vedlitz: Conceived and designed the experiments; Performed the experiments; Contributed reagents, materials, analysis tools or data.

## Data availability statement

The authors do not have permission to share data.

## Additional information

Supplementary content related to this article has been publish online at [URL].

## Declaration of competing interest

The authors declare that they have no known competing financial interests or personal relationships that could have appeared to influence the work reported in this paper.
