# Supplementary material for: Disparities in access and association between access to critical facilities during day-to-day and disrupted access as a result of storm extreme weather events
Source: Heliyon. 2023 Aug 1;9(8):e18841. doi: 10.1016/j.heliyon.2023.e18841 (PMC10412829; doi:10.1016/j.heliyon.2023.e18841)
Supplement: Multimedia component 1 [file mmc1.docx]

The Survey questions supporting the research are presented below.

**Q1. Day-to-day access: About how many minutes does it typically take to travel one way between your home and the closest?**

| **Facilities** | **Minutes** |
| --- | --- |
| Grocery store |  |
| Pharmacy |  |
| Gas station |  |
| Healthcare facility |  |
| Social services |  |
| Place of work |  |
| School |  |

**Q2. Increased travel time: During a past storm-related event in the last 5 years, if available, how many minutes did you need to travel one way to get to an alternative?**

| **Facilities** | **Minutes** |
| --- | --- |
| Grocery store |  |
| Pharmacy |  |
| Gas station |  |
| Healthcare facility |  |

**Q3. Loss of access: During any past floods in the last 5 years, did you lose access to any of the following facilities or services that you used before the flood?**

| **Facilities** | **Minutes** |
| --- | --- |
| Grocery store |  |
| Pharmacy |  |
| Gas station |  |
| Healthcare facility |  |
| Social services |  |
| Place of work |  |
| School |  |

**Q4. Sociodemographic information: Please choose the appropriate answer.**

| **Household subdomain group** | **Survey response options** |
| --- | --- |
| **Income** | Less than $10,000 |
|  | $10,000 to $24,999 |
|  | $25,000 to $49,999 |
|  | $50,000 to $74,999 |
|  | $75,000 to $99,999 |
|  | $100,000 to $149,999 |
|  | $150,000 or more |
| **Ethnicity** | White, non-Hispanic |
|  | Black, non-Hispanic |
|  | Other, non-Hispanic |
|  | Hispanic |
|  | 2+ races, non-Hispanic |
| **MSA Status** | Metro (as defined by U.S. OMB Core-Based Statistical Area) |
|  | Non-Metro |
